# *Notchless* Is Required for Axial Skeleton Formation in Mice

**DOI:** 10.1371/journal.pone.0098507

**Published:** 2014-05-29

**Authors:** Sarah Beck-Cormier, Marie Escande, Céline Souilhol, Sandrine Vandormael-Pournin, Sophie Sourice, Paul Pilet, Charles Babinet, Michel Cohen-Tannoudji

**Affiliations:** 1 Mouse Functional Genetics, Department of Developmental & Stem Cell Biology, Institut Pasteur, Paris, France; 2 Centre National de la Recherche Scientifique, URA 2578, Institut Pasteur, Paris, France; 3 Institut National de la Santé et de la recherche Médicale, U791, LIOAD, STEP group “Skeletal Tissue Engineering and Physiopathology”, Nantes, France; Instituto Gulbenkian de Ciência, Portugal

## Abstract

Maintenance of cell survival is essential for proper embryonic development. In the mouse, *Notchless homolog 1 (Drosophila)* (*Nle1*) is instrumental for survival of cells of the inner cell mass upon implantation. Here, we analyze the function of *Nle1* after implantation using the *Meox2^tm1(cre)Sor^* mouse that expresses the Cre recombinase specifically in the epiblast at E5.5. First, we find that NLE1 function is required in epiblast cells, as *Nle1*-deficient cells are rapidly eliminated. In this report, we also show that the *Meox2^Cre^* transgene is active in specific tissues during organogenesis. In particular, we detect high Cre expression in the vertebral column, ribs, limbs and tailbud. We took advantage of this dynamic expression profile to analyze the effects of inducing mosaic deletion of *Nle1* in the embryo. We show that *Nle1* deletion in this context, results in severe developmental anomalies leading to lethality at birth. Mutant embryos display multiple developmental defects in particular during axial skeletal formation. We also provide evidence that axial defects are due to an increase in apoptotic cell death in the somite at E9.5. These data demonstrate an essential role for *Nle1* during organogenesis and in particular during axial development.

## Introduction

The *Notchless homolog 1 (Drosophila)* (*Nle1*) gene codes for a member of the WD-repeat containing protein family involved in a wide range of cellular functions including cytoskeleton assembly, cell division, transcriptional regulation, RNA processing and signal transduction [1]–[4]. Initially, NLE1 was identified in *Drosophila* as a direct regulator of Notch activity, although the molecular mechanism underlying this regulatory process has not been characterized [2]. Interestingly enough, NLE1 was shown to have an ancient evolutionary origin, appearing before the emergence of pluricellularity and intercellular signaling pathways [5]. In yeast, the NLE1 ortholog Rsa4 is essential for ribosome biogenesis. It assembles in the nucleolus with the pre-60S ribosomal subunit and interacts through its well-conserved amino-terminal region with the AAA-ATPase Rea1. This interaction is required for the disassembly of non-ribosomal factors prior to export of the mature large subunit to the cytoplasm [6]. We recently showed that the key role of NLE1 in 60S biogenesis is conserved during evolution. Using conditional inactivation in adult mice, we demonstrated that NLE1 regulated ribosome biogenesis in hematopoietic stem cells and immature progenitors and was required for the maintenance of these populations [7]. Strikingly, NLE1 was dispensable for ribosome biogenesis, proliferation and differentiation of B lymphocytes, suggesting that alternative pathways for 60S subunit production might exist and be differentially active depending on cell type or degree of differentiation.

Limited data is available so far concerning the role of NLE1 during embryonic development. We previously reported that constitutive *Nle1* inactivation leads to embryonic lethality around the time of implantation due to selective apoptosis of pluripotent cells of the blastocyst [1]. Early embryonic lethality has recently been reported for mice homozygous for non-conservative missense *Nle1* mutations obtained by ENU mutagenesis [8]. To bypass the early embryonic lethality caused by *Nle1* deficiency and address the role of NLE1 following implantation development, we conditionally inactivated the *Nle1* gene using the *Meox2^tm1(cre)Sor^* strain of mice (called MORE hereafter) harboring the *Meox2^Cre^* allele which directs Cre recombinase expression in the epiblast at embryonic day (E) 5.5 [9]. Using this approach, we showed that *Nle1* is required in epiblast cells after implantation. We also uncovered a second wave of transcriptional activity of the *Meox2^Cre^* allele resembling endogenous *Meox2* gene expression profile. Analysis of *Nle1* conditional mutant embryos after gastrulation points to an important role for NLE1 in formation of the axial skeleton.

## Results

### NLE1 is required in epiblast cells after implantation

**Figure 1:**
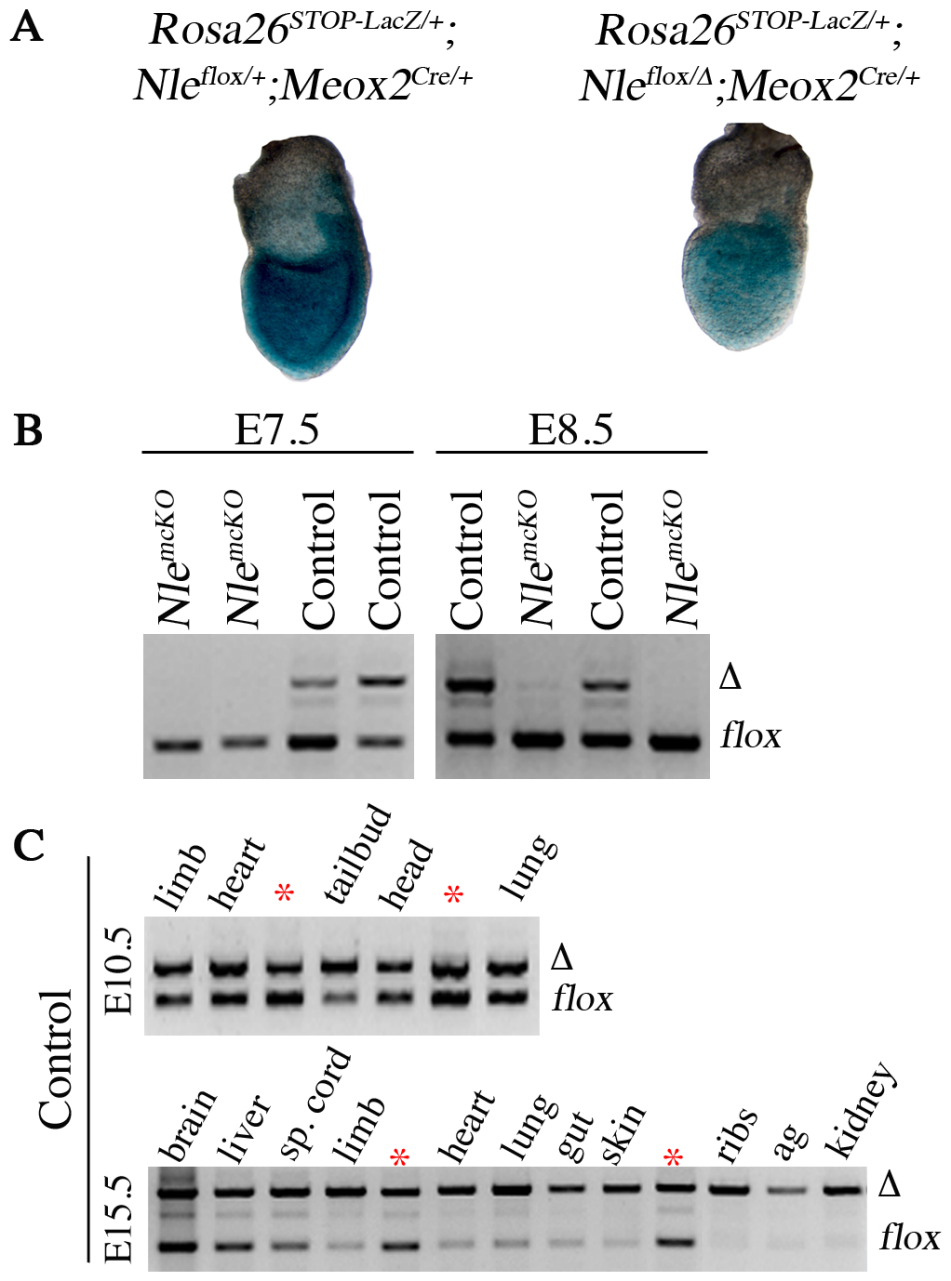
Partial recombination of the *Nle1^flox^* allele by *Meox2^Cre^* and detection of *Nle1*-deficient cells. **A.** At gastrulation, *Meox2^Cre^*-mediated recombination was detected by staining for β-galactosidase activity using *Rosa26^STOP-LacZ^* reporter allele in *Rosa26^STOP-LacZ/+^; Nle1^flox/+^; Meox2^Cre/+^* control embryo (left, n = 3) and in *Rosa26^STOP-LacZ/+^; Nle1^flox/Δ^; Meox2^Cre/+^* mutant embryo (right, n = 3). **B.** PCR amplification of genomic DNA from *Nle1^flox/+^; Meox2^Cre/+^* control embryos showing partial recombination of the *Nle1^flox^* allele at E7.5 (n = 4), E8.5 (n = 12). The recombined (*Nle1* ^Δ^) allele is detected as soon as E7.5. Note that PCR analysis underestimates the recombination efficiency at this stage. Indeed, PCR analysis was performed on whole embryo containing extraembryonic tissues (visceral endoderm, extraembryonic ectoderm) that do not express the Cre recombinase. Presence of *Nle1*-deficient cells was evaluated by PCR amplification of genomic DNA performed on *Nle1^mcKO^* mutant embryos at E7.5 (n = 9) and E8.5 (n = 6). Representative results of PCR are shown. **C.** PCR amplification of genomic DNA from *Nle1^flox/+^; Meox2^Cre/+^* control embryos showing partial recombination of the *Nle1^flox^* allele in several tissues at E10.5 (n = 3) and E15.5 (n = 3). Efficiency of recombination was estimated through comparison with a reference DNA sample (*) derived from a *Nle1^flox/^* ^Δ^ adult mouse. Nested PCR were performed for the E7.5 and E8.5 stages. sp. cord: spinal cord, ag: adrenal glands.

To analyze the function of NLE1 in post-implantation embryos, we adopted a conditional gene targeting strategy. Conditional *Nle1^flox/flox^* mice were crossed to *Nle1^null/+^;Meox2^Cre/+^* mice carrying a *Nle1-null* allele (*Nle1^LacZ^* or *Nle1* ^Δ^) and the *Meox2^Cre^* allele, which drives expression of Cre recombinase in the post-implantation epiblast from E5.5 [1], [9], [10]. We first monitored the activity of the *Meox2^Cre^* allele in our crosses using the *Rosa26^STOP-LacZ^* reporter mice [11]. In E7.5 *Rosa26^STOP-LacZ/+^; Nle1^flox^* ^/+^;*Meox2^cre/+^* control embryos, we observed that Cre was active in the epiblast and the large majority of cells showed recombination at the *Rosa26^STOP-LacZ^* allele (blue cells) as expected (Fig. 1A). Noticeably, the number of blue cells was lower in *Rosa26^STOP-LacZ/+^; Nle1^flox^* ^/Δ^;*Meox2^cre/+^*mutant embryos, suggesting incomplete recombination of the *Nle1^flox^* allele has occurred in the epiblast of mutant embryos. We next monitored the efficiency of recombination at the *Nle1* locus by PCR analysis of whole embryo or dissected embryonic organs at various developmental stages (Fig. 1B–C). The unrecombined *Nle1^flox^* allele was readily detected in E7.5 to E15.5 embryos, indicating that recombination of the *Nle1^flox^* allele in pluripotent epiblast cells was incomplete. Accordingly, *Nle1^LacZ/flox^;Meox2^Cre/+^* mutant embryos were named *Nle1^mcKO^* embryos for *Nle1* mosaic conditional Knock Out embryos since they were composed of a mixture of *Nle1-*deficient (*Nle1^LacZ/^* ^Δ^) and *Nle1-*proficient (*Nle1^LacZ/flox^*) cells.

At E7.5 and E8.5, although the *Nle1^Δ^* allele could be detected in control embryos, it was hardly detectable in *Nle1^mcKO^* embryos (Fig. 1B). No morphological defects were observed in the mutant embryos at these stages suggesting that *Nle1*-deficient cells had been selected at the expense of *Nle1*-proficient cells during gastrulation. The higher proportion of epiblast cells with unrecombined *Rosa26^STOP-LacZ^* allele in mutant embryos compared to controls (Fig. 1A) suggests that selection of cells with low Cre activity had occurred during gastrulation. Importantly, when the Sox2-Cre driver line with higher Cre recombinase activity [12] was used to inactivate *Nle1* in the epiblast cells, developmental arrest at E7.5-E8.5 was observed (Table S1). Collectively, these data indicate that *Nle1* is required for survival and/or proliferation of epiblast cells of postimplantation embryos.

### *Meox2^Cre^* expression is dynamic during embryonic development

**Figure 2:**
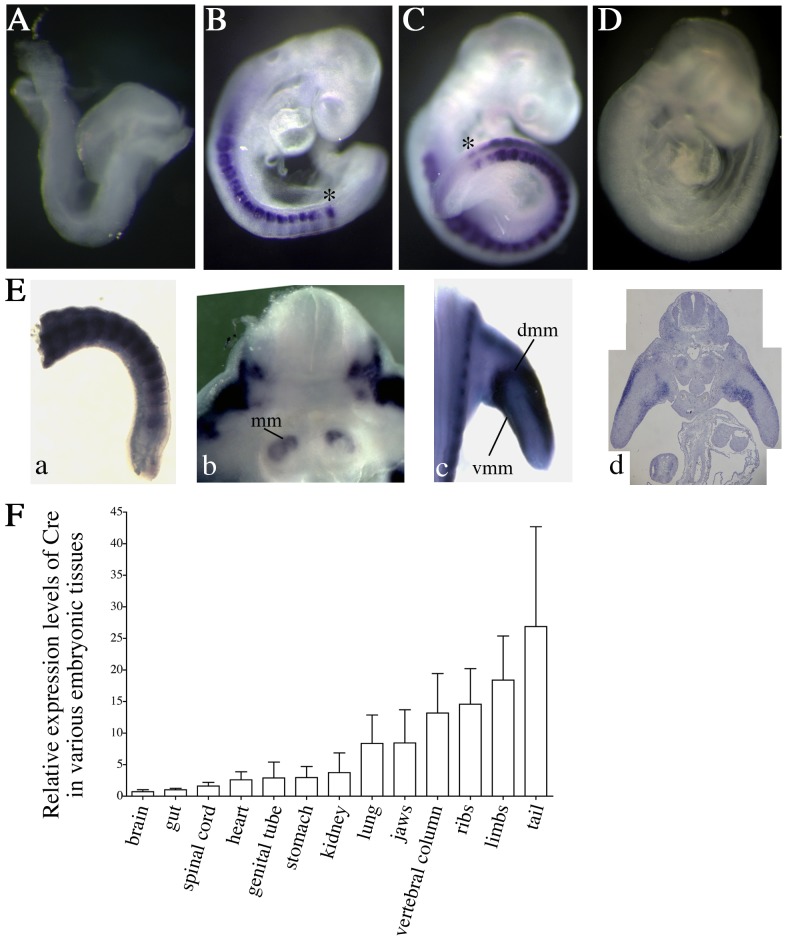
*Meox2^Cre^* expression pattern during post-implantation development. **A–E.** Whole-mount ISH performed on *Meox2^Cre/+^* embryos at E8.5 (A), E9 (B), E9.5 (C) and E11.5 (E). (E8.5: n = 2, E9–E9.5: n = 9, E11.5: n = 2 *Meox2^Cre/+^; Nle1^+/+^* embryos) No signal was observed in *Meox2^+/+^* embryos at any developmental stages (result shown at E9, D). mm: metanephric mesenchyme, dmm and vmm: dorsal and ventral muscle masses, *: anterior part of pre-somitic mesoderm. **F.** Real-time RT-PCR from RNAs extracted from E14.5-organs (n = 3 *Meox2^Cre/+^; Nle1^+/+^* embryos). The expression level of *Cre* in tissues was calculated with the 2^-**ΔΔ**C^_T_ method [42] after normalization with *TBP* and *Tubuline β 5*. We arbitrarily choose to compare *Cre* expression levels in various tissues relative to those expressed in the brain. Bars are means (SD).

At later stages of development, we observed a dynamic profile of recombination at the *Nle1^flox^* allele. In E10.5 control embryos, all tissues displayed similar ratios of *Nle1* ^Δ^ over *Nle1^flox^* allele (∼50% recombination efficiency) except for tailbud samples that contain the pre-somitic mesoderm (PSM) and the newly formed somites, which showed higher degrees of recombination (Fig.1C). Five days later, we observed near complete conversion of *Nle1^flox^* allele to *Nle1* ^Δ^ allele was observed in most tissues except brain, spinal cord and liver (Fig. 1C). This indicated that Cre recombinase expression was not limited to the epiblast and that Cre activity was heterogeneous after gastrulation. The expression profile of the *Meox2^Cre^* allele has been determined using Cre activity reporter lines [12], [13]. Such a strategy allows us to determine the initial phase of Cre recombinase activity in a given lineage but is uninformative regarding the dynamics of Cre expression in the progenies of the primary recombined cells. Consequently, the *Meox2^Cre^* expression profile after gastrulation has not been determined so far. We therefore monitored *Cre* mRNA expression by *in situ* hybridization on E8.5 to E11.5 embryos recovered from crosses between wild-type females and *Meox2^Cre^* ^/+^ males. At E8.5, no *Meox2^Cre^* expression could be observed (Fig. 2A). At E9.0 and E9.5, *Meox2^Cre^* transcriptional activity was detected in the somites and in the anterior PSM (Fig. 2B,C). At E11.5, the *Meox2^Cre^* allele was expressed in the newly formed somites, in limb muscles, metanephric mesenchyme and mesenchymal cells of palatal shelves (Fig. 2E and not shown). In addition, real-time RT-PCR on dissected tissues from E14.5 control embryos confirmed the heterogeneous *Meox2^Cre^* expression pattern showing highest levels in the ribs, limbs, vertebral column and tail (Fig. 2F). We conclude that the MORE mouse strain not only allows recombination of conditional alleles in epiblast cells but also during organogenesis.

### Mosaic inactivation of *Nle1* leads to abnormal organogenesis

**Figure 3:**
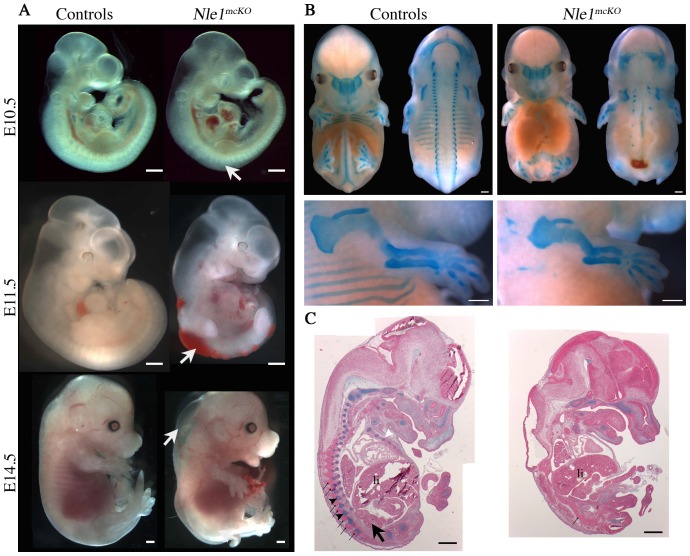
Profound axial anomalies in *Nle1^mcKO^* mutant embryos. **A.** Lateral views of E10.5, E11.5 and E14.5 control and *Nle1^mcKO^* embryos. Irregular somites (E10.5), edema and hemorrhages (E11.5 and E14.5) and vestigial tail (E14.5) are indicated (arrows). **B.** Cartilage staining of E14.5 control (left, ventral and dorsal views) and *Nle1^mcKO^* embryos (right, ventral and dorsal views). Note that ribs are missing and a few vertebrae remnants are detected along the neural tube. Magnified views of upper limbs (bottom) show that appendicular bones are present and exhibit reduced size in mutant embryos. **C.** Alcian blue staining of histological sections of E14.5 control (left) and *Nle1^mcKO^* (right) embryos. Vertebrae (black arrowhead), ribs (white arrowhead) and kidney (arrow) are indicated in the control embryo and are absent in the *Nle1^mcKO^* mutant embryo. Note that dorsal spinal ganglia (thin arrows) are regularly spaced along the vertebral column in control embryos and fused in the *Nle1^mcKO^* mutant embryo. li: liver. Scale bar: 500 µm.

*Nle1^mcKO^* embryos were recovered at expected Mendelian ratios until birth but exhibited multiple and severe developmental anomalies (Table 1 and Fig. 3). Morphological defects were first observed at E10.5 when mutant embryos exhibited segmented but irregular somites (Fig. 3A). From E11.5, all *Nle1^mcKO^* embryos could be distinguished from their littermates by a shortened or absent tail, edema and hemorrhages along the entire length of the embryo (Fig. 3A). To study the formation of the axial skeleton, E14.5 embryos were stained with alcian blue. Analysis of mutant embryos revealed rare axial skeleton structures (absence of ribs, rare and disorganized vertebrae and vestigial tail), whereas cranial bones derived from cephalic mesoderm and neural crest and bones of the limbs derived from lateral mesoderm were present and displayed no gross morphological abnormalities except for a reduced size (Fig. 3B). To be precise about the developmental defects observed in mutant embryos, serial sections were stained with alcian blue. We found, when present, fused vertebrae in mutant embryos compared to controls (Fig. 3C, S1). In addition, *Nle1^mcKO^* embryos exhibited neural tube defects and small or absent kidneys (Fig 3C, S1). At E18.5, alcian blue (cartilage) and alizarin red (bone) staining of skeletal preparations showed that, in addition to the absence of axial skeleton, the elements of cranial and appendicular skeleton (limbs) were undersized and showed scarce mineralized bone tissue in *Nle1^mcKO^* embryos compared to controls (Fig S2). This phenotype was also evidenced at E17.5 using 3D µCT images acquisitions from E17.5 control and mutant embryos (Movies S1, S2). These results showed that *Nle1* is essential during organogenesis and in particular for the formation of the axial skeleton.

**Table 1:** Number of mutant embryos and pups obtained from crosses between *Nle1^flox/flox^* and *Nle1^LacZ +/^; Meox2^Cre/+^* mice at various stages.

Stages	Total embryos	Mutant progeny^a^
E6.5–E8.5	203	50 (25%)
E9.5–E11.5	364	104 (29%)
E13.5–E15.5	56	19 (34%)
E17.5–E18.5	48	13 (27%)
pups	41	11 (27%)^b^

a Absolute number and frequency (%).

b No mutant pups were found alive at birth.

### *Nle1^mcKO^* mutant embryos have impaired somite formation

**Figure 4:**
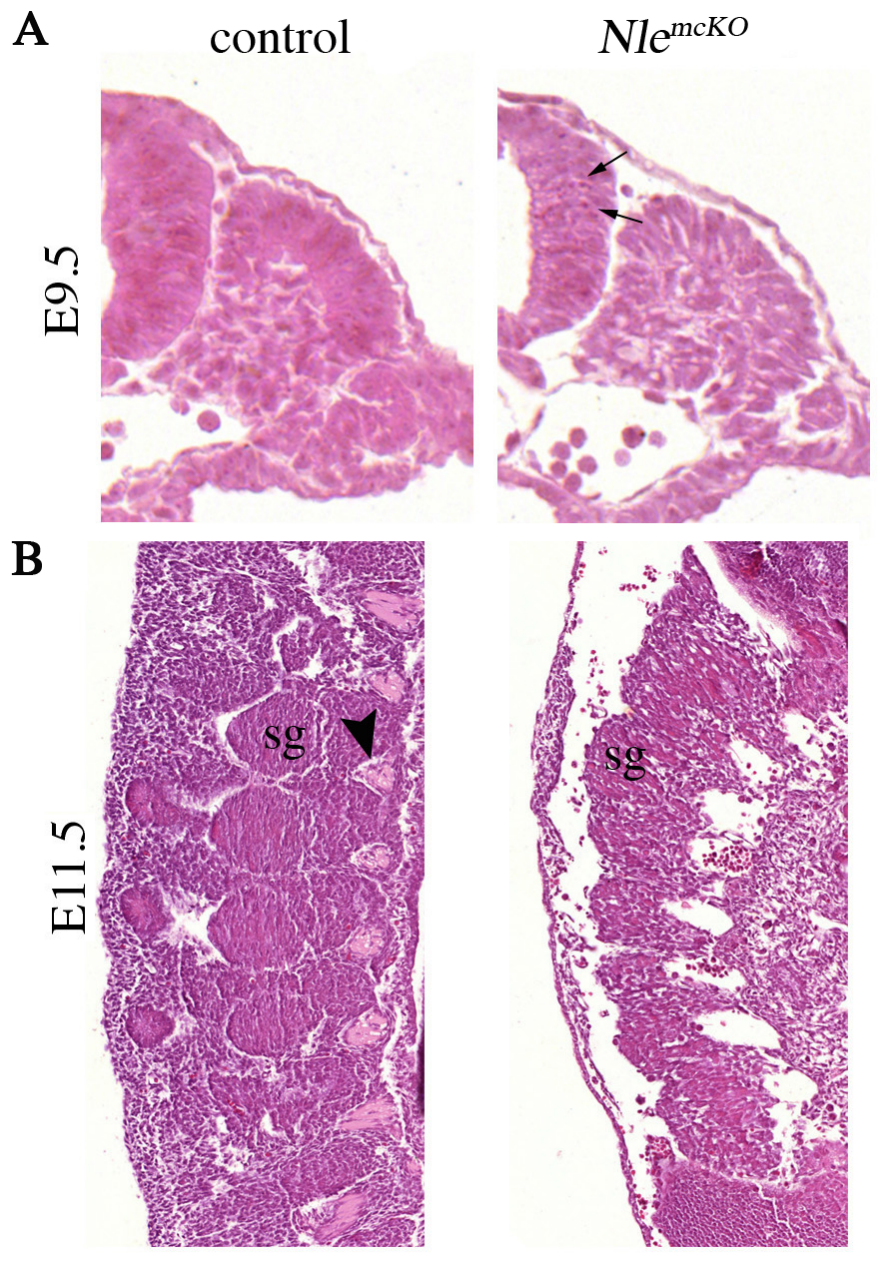
Histological analysis of somitic regions of control and *Nle1^mcKO^* mutant embryos. Embryos were sectioned and stained with hematoxylin and eosin at E9.5 (transverse section of the tail, **A**) and at E11.5 (sagittal section of interlimb region, **B**). Arrow indicated the picnotic nuclei visible in the neural tube of E9.5 *Nle1^mcKO^* embryos. At E11.5, absence of sclerotomal condensations (arrowhead in control) and fusion of dorsal spinal ganglia (sg) are shown.

**Figure 5:**
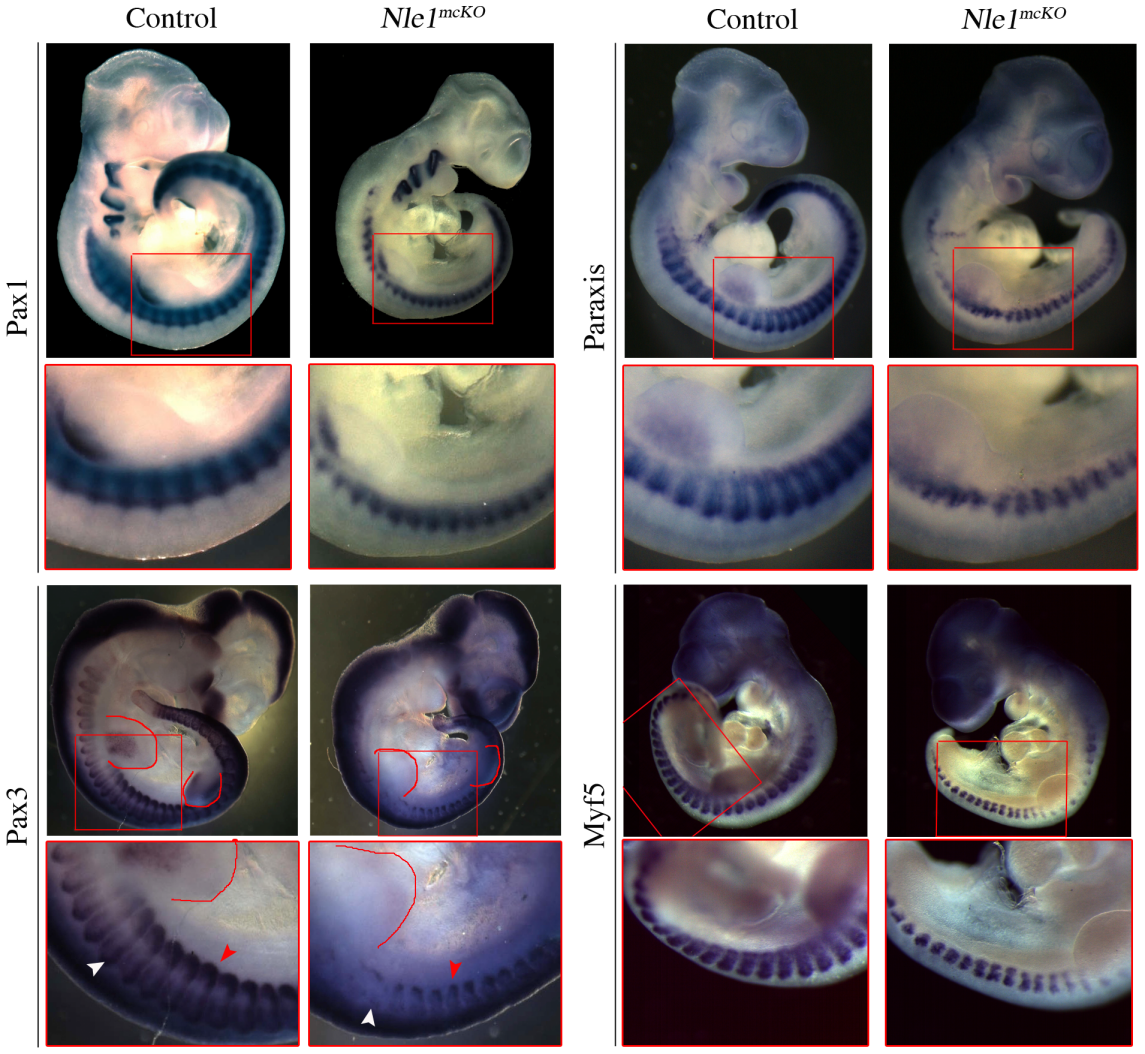
Expression pattern of markers for somitic lineages in E10.5 control and *Nle1^mcKO^* embryos. Whole-mount *in situ* hybridizations were performed with *Pax1*, *Paraxis*, *Pax3* and *Myf5* riboprobes (n = 3 control, n = 3 *Nle1^mcKO^* mutant embryos for *Pax1*, *Paraxis* and *Pax3*, n = 2 control and n = 2 control, n = 2 *Nle1^mcKO^* mutant embryos for *Myf5*. Pax3 is expressed in the dorsomedial (white arrowhead) and dorsolateral (red arrowhead) lips of the dermomyotome in E10.5 control embryos. The red line indicate the limb bud where *Pax3*-positive muscle progenitors are visible in the control embryo but not in the *Nle1^mcKO^* embryo. Red boxes mark an embryonic region shown enlarged below each embryo.

The observation that axial skeleton development is compromised in *Nle1^mcKO^* mutant embryos led us to analyze somitogenesis. After segmental border formation and epithelialization, cells located in the dorsal region of the somite give rise to dermomyotome and myotome, while cells in the ventro-medial region de-epithelialize to form the sclerotome responsible for the future axial skeleton. To characterize the nature of axial defects, we first performed histological analysis of somitic region of control and *Nle1^mcKO^* embryos between E9.5 (stage at which *Meox2^Cre^* is active again) and E11.5. At E9.5, while dermomyotome of *Nle1^mcKO^* somites was clearly visible on transverse sections, both the dermomyotome and the underlying mesenchyme appeared as loose tissues compared to controls (Fig. 4A). Sagittal sections of E11.5 *Nle1^mcKO^* embryos revealed fusion of the dorsal root ganglia and absence of sclerotome condensations that are indicative of abnormal somitogenesis (Fig. 4B). To further precise the origins of axial skeleton defects, we analyzed the expression pattern of somitic markers by whole mount *in situ* hybridization at E9.5 and E10.5. We observed that the expression pattern of markers of all somitic compartments (*Paraxis* for newly formed somites, *Pax1* for sclerotome, *Paraxis* and *Pax3* for the dermomyotome and *Myf5* for the myotome) were similar in *Nle1^mcKO^* embryos compared to controls at E9.5 (Fig. S3). At E10.5, *Pax1* was downregulated in the dorsal part of somites and especially in the most rostral somites in *Nle1^mcKO^* embryos (Fig. 5). This downregulation was specific for sclerotomal defects since normal *Pax1* expression was observed elsewhere (pharyngeal arches and at the basis of the limb bud). We also examined the expression pattern of dermomyotome and myotome markers (*Paraxis*, *Pax3* and *Myf5*) and showed that both compartments were perturbed in E10.5 *Nle1^mcKO^* embryos. Indeed, *Paraxis* exhibited a segmental pattern in the somites of control embryos, whereas expression in *Nle1^mcKO^* embryos was truncated dorsally with a more severe downregulation in the most rostral somites. In addition, no expression of *Myf5* was detected in the dorsal part of the mutant somites. Similarly, *Pax3* expression domain in the dermomyotome was severely reduced, especially in the dorsomedial lips, whereas a narrow signal was still observed in the dorsolateral lips. Absence of *Pax3* expression was observed in the limb muscle primordial cells. In contrast, no major alterations of *Pax3* expression profile was observed in the central nervous system. These data show that *Nle1* function is critically required for the patterning of somitic derivatives.

### *Nle1^mcKO^* mutant embryos exhibit upregulated caspase3-dependent apoptosis in the somites and the neural tube

**Figure 6:**
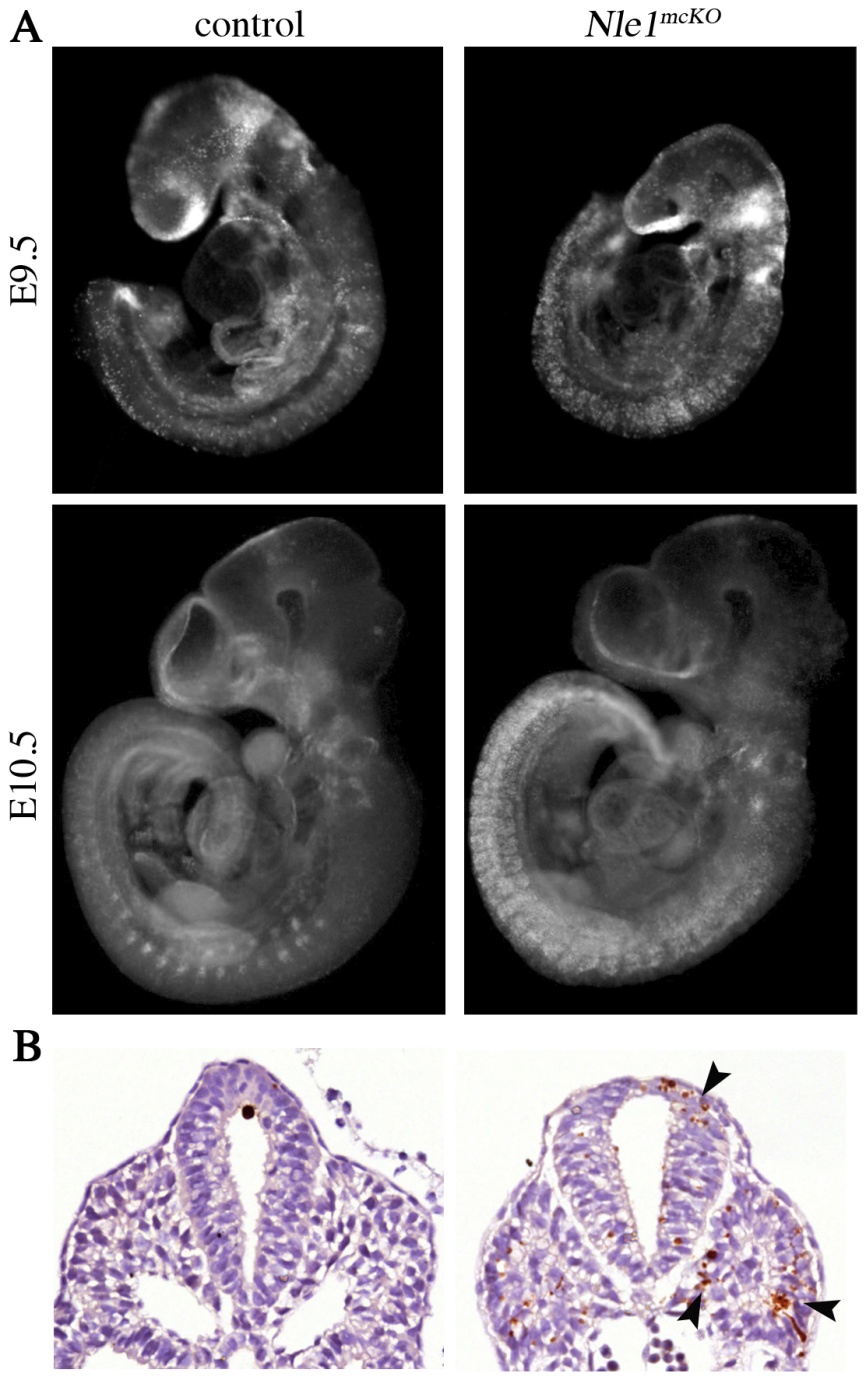
Increased cell death is observed in *Nle1^mcKO^* mutant embryos. **A.** E9.5 and E10.5 control and mutant embryos were incubated in LysoTracker Red solution. Normal developmental apoptosis was observed in the head (E9.5 and E10.5) and in the ventral part of the somites (E10.5) of control embryos. In *Nle1^mcKO^* embryos, apoptosis in the head was similar as control embryos, whereas a marked increase in apoptosis was observed in the neural tube and the entire somites at E9.5 (n = 2 mutants and n = 6 controls including 2 *Meox2^Cre^* embryos) and E10.5 (n = 3 mutants and n = 8 controls including 2 *Meox2^Cre^* embryos). **B.** Immunostaining for the active form of caspase3 protein in E9.5 control and *Nle1^mcKO^* embryos. Representative results of immunostaining of transverse section of the caudal part of embryos are shown. In *Nle1^mcKO^* embryos, high number of apoptotic cells was observed (arrowheads) in the neural tube and the somites. (n = 3 mutants and n = 4 controls including a *Meox2^Cre^* embryo). No increase in apoptosis was observed due to the expression of the *Meox2^Cre^* allele.

The observation that all somitic markers are rapidly downregulated in *Nle1^mcKO^* embryos led us to analyze if a loss of somitic cells had occurred due to cell death. Cell death was investigated by immunostaining using a specific antibody recognizing the active form of caspase 3 and by using LysoTracker^®^ Red marker, previously demonstrated to be an accurate marker of cell death in embryos [14], [15]. Importantly, Cre-mediated apoptosis has been described in several Cre transgenic mouse lines [14], but so far not in the *Meox2^Cre^* line. Accordingly, we didn't observed an increase in cell death in Cre-expressing (*Nle1^LacZ/+^; Meox2^Cre/+^*) control embryos compared to non expressing (*Nle1^LacZ/+^* or *Nle1^flox/+^*) ones (data not shown). In both types of control embryos, apoptosis was detected essentially in the head and in the ventral part of the mature somites (Fig. 6A, movie S3 and S5). In contrast, a dramatic increase in cell death was observed in the entire somites and in the neural tube from *Nle1^mcKO^* embryos at E9.5 and E10.5 (Fig. 6 and S4, movie S4 and S6). In the neural tube, upregulated apoptosis was detected caudally to the forelimb but not rostrally where caspase3-positive signals were equivalent to those observed in control embryos (Fig. S4). In the somites, upregulation of apoptosis was observed both in newly formed and mature somites. Apoptosis levels in other tissues were indistinguishable in mutant and control embryos. Of note, no increase in apoptosis was observed at E7.5, which is consistent with the “wild-type like” genotype of the *Nle1^mcKO^* embryos at these stages (not shown). Altogether, these results suggest that downregulation of *Nle1* in post-implantation embryos leads to upregulation of apoptosis in the somites and the neural tube that probably contributes to abnormal somitogenesis and profound axial defects.

## Discussion

In the present study, the use of the *Meox2^Cre^* line shows that NLE1 is critically required during organogenesis and in particular for the axial skeleton formation. Based on our data, we propose that partial disruption of *Nle1* leads to an increase in apoptotic cell death in the somites responsible for abnormal patterning of the somites leading to axial skeleton defects.

The MORE mouse strain is commonly used to generate null alleles from conditional floxed alleles or to determine if defects in extraembryonic tissues contribute to mutant phenotype by invalidating the gene of interest specifically in the epiblast [9]. The efficiency of recombination mediated by *Meox2^Cre^* depends on the floxed allele to be recombined. Hence, *Meox2^Cre^* activity allowed the full recombination of a floxed *Retinoblastoma* gene and the generation of *Rb*-deficient mice [16]. In contrast, *Meox2^Cre^* activity has been shown to mediate only partial recombination of the conditional alleles of *Sonic Hedgehog*, *Bmpr1a*, *Nodal*, *Pcsk5*, *PPARγ* and *fgf8* genes during development [12], [13], [17]–[20]. Here, we show that *Meox2^Cre^* activity in the post-implantation epiblast was not sufficient to achieve full recombination of the *Nle1^flox^* allele as indicated by the fact that *Nle1^flox^* allele can be readily detected in E7.5 and E8.5 control embryos. At these stages, mutant embryos were almost exclusively composed of unrecombined cells, and we propose that *Nle1*-deficient epiblast cells are eliminated rapidly after their generation. Accordingly, when using a more active epiblast Cre driver line, i.e the Sox2-Cre line, embryonic lethality around E7.5 was observed. Collectively, our data demonstrate that the use of the *Meox2^Cre^* line does not allow the generation of embryos deficient for *Nle1* prior to organogenesis despite Cre recombinase expression in the epiblast. While the MORE mouse has proven its interest in generating loss-of-function phenotypes in the embryo-proper, caution should be taken when using this strain to study gene function in early embryos. Indeed, in case of genes with essential functions for epiblast cells, low efficiency of recombination combined to rapid expansion of non-recombined epiblast cells may give rise to misleading interpretations.

The MORE line was obtained by inserting a nls-Cre recombinase cassette into the *Meox2* gene by homologous recombination [9]. Cre activity was thus anticipated to mimic the expression profile of the endogenous *Meox2* gene, which starts to be expressed in the epithelial somite and then becomes restricted to the sclerotome and limb musculature [21], [22]. Unexpectedly and for reasons that have remained unexplained so far, the *Meox2^Cre^* allele drives Cre expression in the epiblast at E5.5. The observation that the *Nle1^flox^* allele was recombined in a tissue-specific manner during the second half of gestation lead us to unravel a second wave of transcriptional activation of the *Meox2^Cre^* allele that starts between E8.5 and E9.5 and is restricted to the somites and the anterior part of the PSM. At later stage of development, *Meox2^Cre^* expression was detected in various organs, being low in endoderm-derived (liver, gut) and neurectoderm (brain, spinal cord) tissues and high in mesoderm-derived (ribs and vertebral column, heart) tissues. Although our analysis at late stages of development was performed by real-time RT-PCR and therefore lacks cellular resolution, *Meox2^Cre^* expression seems consistent with the widespread expression of the *Meox2* gene in mesenchymal derivatives of E14.5 embryos [23]. Our data thus suggests that after a transient peak of expression in epiblast cell of early postimplantation embryos, *Meox2^Cre^* expression is likely to recapitulate endogenous *Meox2* gene expression. It would be interesting to determine whether this secondary wave of Cre expression contributed to the axial skeleton-specific phenotype previously reported following forced *Cyclooxygenase-2* expression using the MORE line [24].

One of the most striking abnormalities of *Nle1^mcKO^* embryos is the lack of axial skeleton. By the time the second wave of *Meox2^Cre^* expression, a dramatic increase in apoptosis was observed in the somites of E9.5 mutant embryos that likely contribute to somite mispatterning. Consistent with abnormal axial skeleton formation, histological and gene expression analyses showed that the sclerotome compartment was severely disorganized and that Pax1 was downregulated at E10.5. At E11.5, histological sections confirmed the dramatic effect of *Nle1* invalidation on the sclerotomal compartment since no chondrogenic differentiation was observed. Other defects were observed in *Nle1^mcKO^* embryos including kidney dysgenesis, neural tube defects, edemas (probably due to cardiac defects [25], [26]) and hemorrhage. During embryonic development, *Meox2* is expressed in the kidney (http://www.gudmap.org), heart and vasculature [27]–[29]. *Nle1* inactivation may therefore be directly responsible for the defects observed in these tissues. In contrast, the causes for increased apoptosis in the neural tube of *Nle1^mcKO^* embryos at E9.5 and E10.5 are unclear. Indeed, no *Cre* expression was detected in the neural tube at these stages. An intriguing possibility would be that apoptosis of neural cells results from a cell non-autonomous process following *Nle1* inactivation in the adjacent somitic compartment.

In Drosophila, NLE1 has been proposed to be a direct regulator of the Notch signaling pathway [2]. Since, the Notch pathway is known to tightly regulate somitogenesis in vertebrates [30], [31], abnormal Notch signaling could be a plausible explanation for the failure in somite patterning observed in the *Nle1^mcKO^* embryos. However, the somitic defects reported for embryos mutant for several members of the Notch pathway differ from those observed in *Nle1^mcKO^* embryos. Hence, inactivation of *Notch1*, *Dll1* or *RBPJκ* genes lead to abnormal timing and coordination of somite segmentation, highlighted by disruptions in size and bilateral symmetry of the somites [32]–[34]. By contrast, *Nle1^mcKO^* somites were correctly segmented as judged from histological examinations and segmented expression of somitic markers. Moreover, absence of somites at the caudal most regions was also reported in embryos mutant for several members of the Notch pathway [32], [34]–[36], while this phenotype was not observed in *Nle1^mcKO^* embryos. While one should keep in mind that conditional inactivation of *Nle1* by *Meox2^Cre^* is not temporally, spatially or quantitatively equivalent to the mentioned Notch mutants, the phenotypic discrepancies suggest that abnormal somite patterning in *Nle1^mcKO^* embryo is not caused by a dysregulation of the Notch pathway. In yeast, NLE1 ortholog, Rsa4, was shown to participate in pre-60S ribosomal subunit assembly [6], and conservation of the critical role of NLE1 in ribogenesis was recently demonstrated in mouse embryonic stem cells and immature hematopoietic cells in adult mice [10]. Defective ribosome biogenesis following *Nle1* inactivation was shown to trigger the disappearance of hematopoietic stem cells and immature progenitors through p53-dependent mechanisms. It is therefore likely that the phenotypes observed in *Nle1^mcKO^* embryo were consecutive to defects in ribosome biogenesis. Interestingly, defects in the craniofacial, axial and limb skeleton were reported in patients and animals models with haploinsufficiency for ribosomal protein genes or mutations for ribosome biogenesis factors [37]–[40]. However, limited data are available concerning the mechanisms by which these mutations are affecting the developing skeleton. Strikingly, haploinsufficiency of ribosomal protein L38 (RPL38) in mice lead to abnormal axial skeletal patterning due to selective reduction in the translation of a subset of *Hox* mRNAs [41] indicating that ribosome composition may also impinges on developmental processes. Interestingly, outcompetition between wild-type and Rpl24^+/−^ cells is observed in Rpl24^+/−^ mutant mice [37], reminiscent of the selection of *Nle1*-deficient cells at the expense of *Nle1*-proficient cells that we observed in *Nle1^mcKO^* embryos before gastrulation. Further studies will be required to establish whether ribogenesis is affected in *Nle1^mcKO^* embryos and to determine the mechanisms responsible for the elimination or outcompetition of *Nle1-*deficient cells.

## Materials and Methods

### Ethics statement

Animals were housed in the Institut Pasteur animal facilities accredited by the French Ministry of Agriculture to perform experiments on live mice (accreditation B 75 15–06, issued on May 22, 2008) in appliance of the French and European regulations on care and protection of the Laboratory Animals (EC Directive 86/609, French Law 2001–486 issued on June 6, 2001). Protocols were approved by the veterinary staff of the Institut Pasteur animal facility and were performed in compliance with the NIH Animal Welfare (Insurance #A5476–01 issued on 02/07/2007).

### Mice

To specifically inactivate *Nle1* in the epiblast, *Nle1* ^LacZ/+^ mice [1] were crossed to *Meox2^Cre/+^* [9] to produce *Nle1^LacZ/+^;Meox2^Cre/+^* mice. These mice were then crossed with conditional *Nle1^flox/flox^* mice [10] to produce control and *Nle1^mcKO^* embryos. International strain nomenclature are *Nle1^tm1Cba^* for *Nle1^LacZ^* strain, *Nle1^tm1.1Cota^* for *Nle1^flox^* strain and *Meox2^tm1(cre)Sor^* for the *Meox2^Cre/+^* strain. The colonies were maintained on a 129Sv/C57BL/6 mixed genetic background. *Nle1^mcKO^* mutant embryos were systematically compared to control littermates. Genotyping of embryos was then performed by PCR using the following primers LacZ-Forward: 5′- ACTATCCCGACCGCCTTACT -3′; LacZ-Reverse: 5′- GCTGGTTTCCATGAGTTGCT -3′; Cre-Forward: 5′- CACGACCAAGTGACAGCAAT -3′ and Cre-Reverse: 5′- TCCCCAGAAATGCCAGATTA -3′. Female mice were used between 6 and 14 weeks of age for most of the experiments. Pregnant mice were sacrificed by cervical dislocation for embryo recovery.

### PCR genotyping of embryos, foetal tissues and mice

Biopsies from mice and portion of embryos (i.e., egg cylinders, yolk sacs) were used for genotyping by PCR. These tissues were lysed in 50 mM Tris-Hcl pH 8.5, 100 mM NaCl, 0,5% Tween and 0,1 mg/ml proteinase K at 56°C. Proteinase K was then inactivated at 95°C for 15 min before PCR amplification.

### Real-time RT-PCR

Total RNA was isolated from organs of E14.5 Meox2^Cre/+^ embryos using Nucleospin RNA columns (Macherey Nagel). RT-PCR amplifications were performed using SuperScript III First-Strand Synthesis kit (Invitrogen) according to the manufacturer's instructions. Real time PCR was performed using SYBR Select Master Mix (Life Technologies) on an Mx3000P detection system (Agilent Technologies). The *TBP* and *Tubuline β 5* genes were used as reference genes and expression differences were calculated as described [42]. Primers used are: Cre-Forward: 5′- CACGACCAAGTGACAGCAAT -3′ and Cre-Reverse: 5′- TCCCCAGAAATGCCAGATTA -3′, TBP-Forward: 5′-AGAACAATCCATACTAGCAGC-3′ and TBP-Reverse: 5′-GGGAACTTCACATCACAGCTC-3′, and Tubulineβ5-Forward: 5′-GATCGGTGCTAAGTTCTGGGA-3′ and Tubulinβ5-Reverse: 5′-AGGGACATACTTGCCACCTGT-3′.

### Histological analysis

Embryos were fixed overnight in 4% paraformaldehyde and then dehydrated and embedded in paraffin. Next, 4 µm sections were stained with hematoxylin and eosin or alcian blue using standard procedures.

### Skeletal preparations

For staining and visualization of whole skeletons, embryos were dissected and stained with alizarin red S and alcian blue 8GX (Sigma) as described [43]

### 3D- µCT image acquisitions

E17.5 embryos were collected, fixed in formol. Embryos were imaged using X-ray radiation micro-CT (SkyScan 1072). A microfocus X-ray tube was used as a source (50 kV, 173 µA). The specimen was mounted on a turntable that could be shifted automatically by 180° with a rotation step of 0.45° in the axial direction. The “SkyScan 1072” system provides an image pixel size of 18.88 µm. X-ray images were transformed by NRecon software (skyscan).

### Whole-mount *in situ* hybridization

*In situ* hybridization was carried out as described [44]. Two to five embryos were used for each marker and stage. Antisense probes used during this study were *Paraxis*, *Pax1*, *Pax3*, *Myf5* (provided by S. Tajbakhsh). *Cre* riboprobe was generated from PCR amplification of *Cre* cDNA by using specific primer for the *Cre* flanked by T7 and T3 promoters: Cre-T3Forward, 5′-GAGAATTAACCCTCACTAAAGGGGGACATGTTCAGGGATCGCCAGGCG and Cre-T7rev, 5′-GAGTAATACGACTCACTATAGGGTATTTACATTGGTCCAGCCACCAG.

Photographs of whole-mount stained embryos were taken with the SMZ 1500 stereomicroscope (Nikon) with the AxioCam HRc camera (Zeiss).

### Cell death analysis

Cell death was detected by incubating whole embryos in 5 µM LysoTracker Red (Invitrogen L7528) in Hank's balanced salt solution for 30 min at 37°C in 5% CO_2_ in air. Embryos were then washed in phosphate buffered saline (PBS), dehydrated in methanol, and cleared in 1∶2 benzyl alcohol: benzyl benzoate. Photographs of stained embryos were taken with an inverted microscope Zeiss Axiovert 200M with a Zeiss apotome system controlled by the Zeiss axiovision 4.4 software. The CCD camera used was a Roper Scientific Coolsnap HQ. Immunohistochemistry using antibody against activated-caspase3 (Asp175; Cell Signalling) were used according to the manufacturer's instructions. Whole-mount immunochemistry was performed on E7.5 embryos using antibody against human activated caspase-3 (1∶200; Pharmingen) as described [45].

### X-Gal staining

After fixation of embryos, β-galactosidase expression was visualized by staining with 5-bromo-4-chloro-3-indolyl-β-D-galactopyranoside (X-Gal for embryos; Life Technologies).

## Supporting Information

Figure S1 **Spinal cord and kidney anomalies in** ***Nle1^mcKO^*** **mutant embryos.** **A.** Hematoxylin-eosin staining of histological section of E14.5 control (upper panel) and *Nle1^mcKO^* (lower panel) embryos. On sagittal sections, fused and rare cartilage primordium of spinal column is indicated (arrowheads). Edema (asterisks) and dilated central canal of the spinal cord (sp) are clearly visible. Kidneys (arrow) are indicated in the control embryos and in the *Nle1^mcKO^* mutant embryos (sagittal sections). li: liver, s: stomach, v: vertebra. Scale bars: 500 µm.
(TIF)

Figure S2 **Alcian blue/alizarin red S double staining of the skeleton of E18.5 embryos.** **A.** Whole skeleton staining of *Nle1^mcKO^* (left) and control (right) embryos. **B.** Magnification on the forelimb and humerus. Long bones of *Nle1^mcKO^* embryos are smaller and present a significant delay of mineralization.
(TIF)

Figure S3 **Expression pattern of markers for somitic lineages in E9.5 control and** ***Nle1^mcKO^*** **embryos.** Whole-mount *in situ* hybridizations were performed with *Pax1, Paraxis*, *Pax3* and *Myf5* riboprobes (n = 4 control, n = 3 *Nle1^mcKO^* mutant embryos for *Pax1*, n = 4 control, n = 5 *Nle1^mcKO^* mutant embryos *Paraxis*, n = 4 control, n = 4 *Nle1^mcKO^* mutant embryos for *Pax3*, n = 3 control, n = 6 *Nle1^mcKO^* mutant embryos for *Myf5*. No difference between *Nle1^mcKO^* and control embryos was observed for any marker tested.
(TIF)

Figure S4 **Analysis of apoptosis in E9.5 control and** ***Nle1^mcKO^*** **embryos.** Immunostaining for the active form of caspase3 protein at E9.5 in control (left) and *Nle1^mcKO^* (right) embryos are shown. Embryos were embedded in agarose before being embedded in paraffin. Serial transverse sections are shown. In control embryos, apoptotic cells (arrowhead) were observed in the epidermis, the neural tube and otic pit. In *Nle1^mcKO^* embryos, an abnormally high number of apoptotic cells was observed in the neural tube caudally to the forelimb and in the epithelial and mature somites. Black arrowheads indicate upregulated apoptosis and red arrowheads indicate normal developmental apoptosis (n = 3 mutants and n = 4 controls including a *Meox2^Cre^* embryo). ba: branchial arch, cr: caudal region of embryo, he: heart, ne: neuroepithelium, nt: neural tube, ot: otic vesicle, op: optic vesicle, so: somite.
(TIF)

Table S1 **Number of mutant embryos obtained from crosses between** ***Nle1^flox/flox^*** **and** ***Nle1*** **^Δ/+^;** ***Sox2^Cre/+^*** **mice at various embryonic stages.**
(DOCX)

Movie S1 **MicroCT three-dimensional images of E17.5 control embryo.**
(MOV)

Movie S2 **MicroCT three-dimensional images of E17.5** ***Nle1^mcKO^*** **embryo showing absence of ribs and vertebrae.**
(MOV)

Movie S3 **Cell death analysis in E9.5 control embryo.** Representative image of cell death status in one E9.5 control embryo. Normal developmental apoptosis is observed in the head.
(MOV)

Movie S4 **Cell death analysis in E9.5** ***Nle1^mcKO^*** **embryo.** Representative image of cell death status in one E9.5 mutant embryo. Apoptosis in the head was similar as control embryos, whereas a severe increase in apoptosis is observed in the neural tube and the entire somites.
(MOV)

Movie S5 **Cell death analysis in E10.5 control embryo.** Representative image of cell death status in one E10.5 control embryo. Normal developmental apoptosis is observed in the head and in the ventral part of the somites.
(MOV)

Movie S6 **Cell death analysis in E10.5** ***Nle1^mcKO^*** **embryo.** Representative image of cell death status in one E10.5 *Nle1^mcKO^* embryo. Apoptosis in the head was similar as control embryos, whereas a severe increase in apoptosis is observed in the neural tube and the entire somites.
(MOV)
