# Supplementary figures and images for: Notchless Is Required for Axial Skeleton Formation in Mice
Source: PLoS One. 2014 May 29;9(5):e98507. doi: 10.1371/journal.pone.0098507 (PMC4038589; doi:10.1371/journal.pone.0098507)

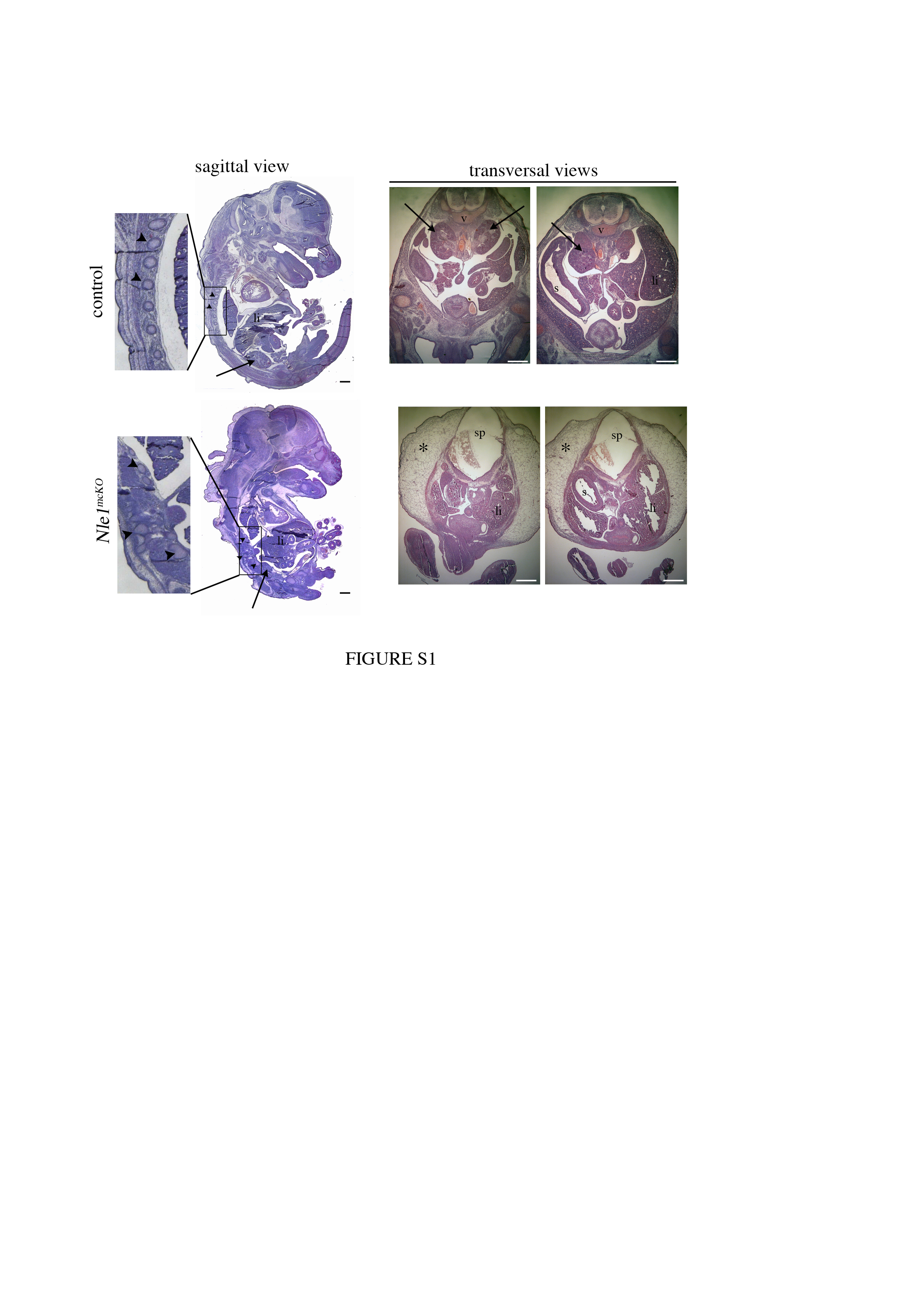

Supplement: Figure S1 — Spinal cord and kidney anomalies in Nle1mcKO mutant embryos. A. Hematoxylin-eosin staining of histological section of E14.5 control (upper panel) and Nle1mcKO (lower panel) embryos. On sagittal sections, fused and rare cartilage primordium of spinal column is indicated (arrowheads). Edema (asterisks) and dilated central canal of the spinal cord (sp) are clearly visible. Kidneys (arrow) are indicated in the control embryos and in the Nle1mcKO mutant embryos (sagittal sections). li: liver, s: stomach, v: vertebra. Scale bars: 500 µm. (TIF) [file pone.0098507.s001.tif]

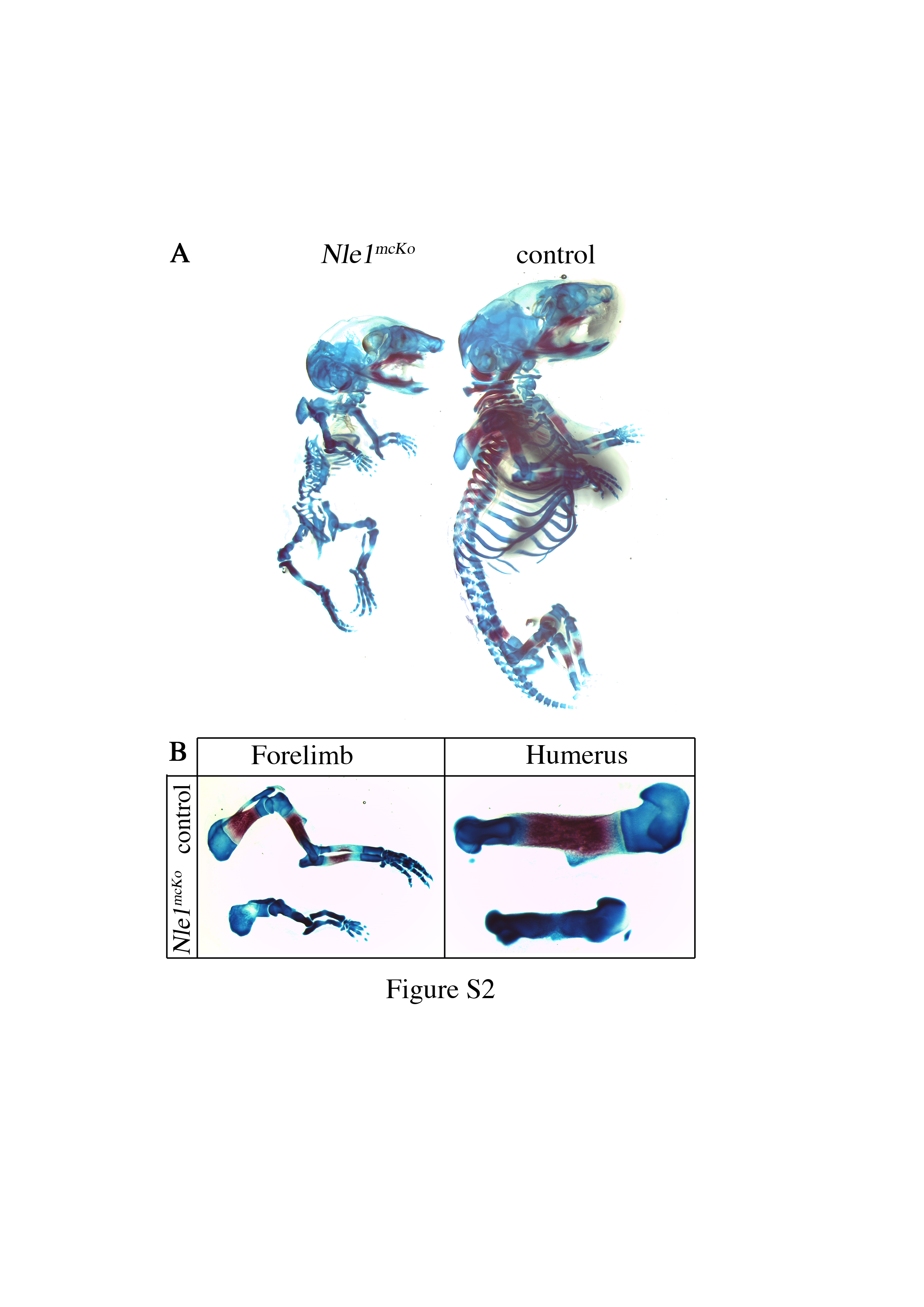

Supplement: Figure S2 — Alcian blue/alizarin red S double staining of the skeleton of E18.5 embryos. A. Whole skeleton staining of Nle1mcKO (left) and control (right) embryos. B. Magnification on the forelimb and humerus. Long bones of Nle1mcKO embryos are smaller and present a significant delay of mineralization. (TIF) [file pone.0098507.s002.tif]

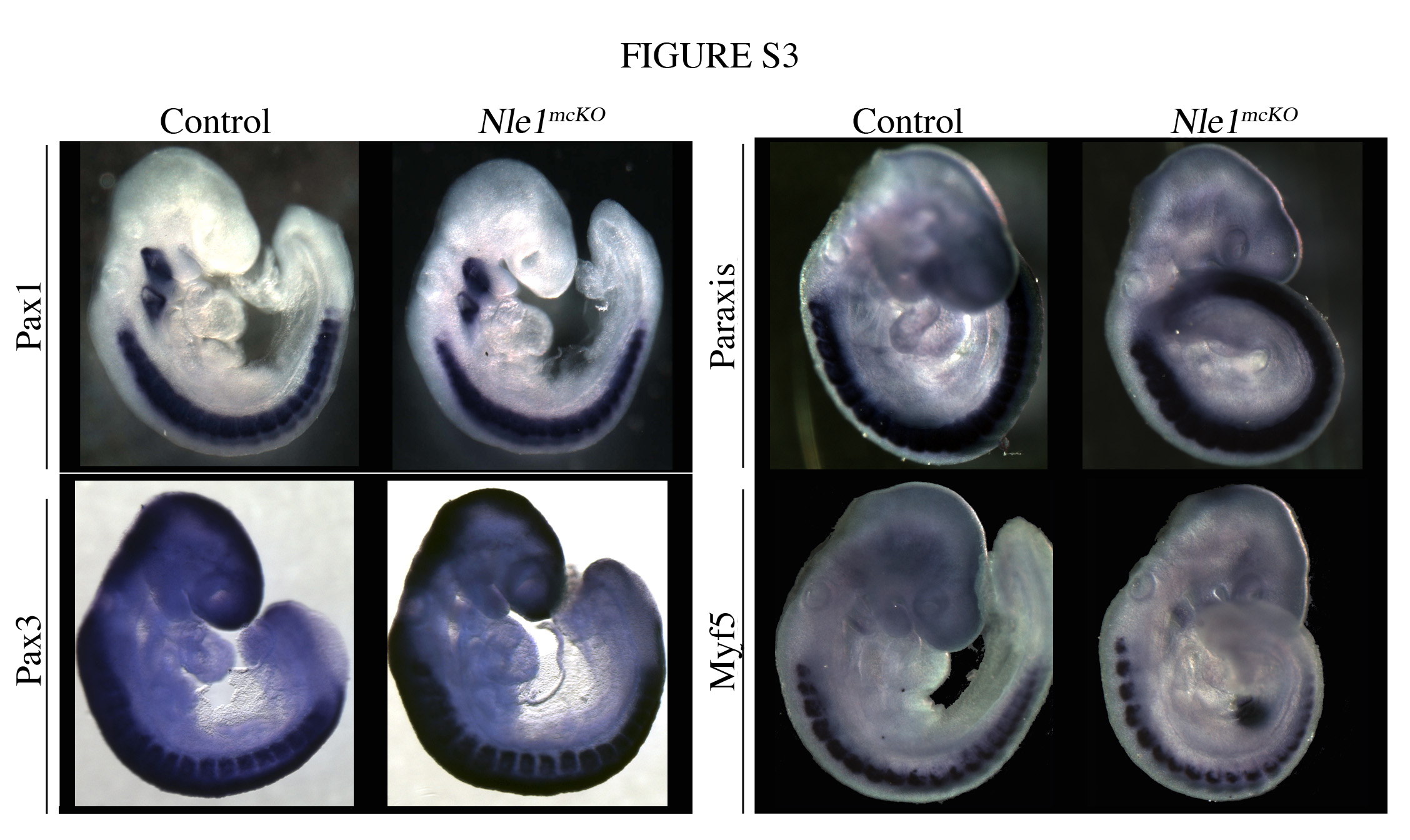

Supplement: Figure S3 — Expression pattern of markers for somitic lineages in E9.5 control and Nle1mcKO embryos. Whole-mount in situ hybridizations were performed with Pax1, Paraxis, Pax3 and Myf5 riboprobes (n = 4 control, n = 3 Nle1mcKO mutant embryos for Pax1, n = 4 control, n = 5 Nle1mcKO mutant embryos Paraxis, n = 4 control, n = 4 Nle1mcKO mutant embryos for Pax3, n = 3 control, n = 6 Nle1mcKO mutant embryos for Myf5. No difference between Nle1mcKO and control embryos was observed for any marker tested. (TIF) [file pone.0098507.s003.tif]

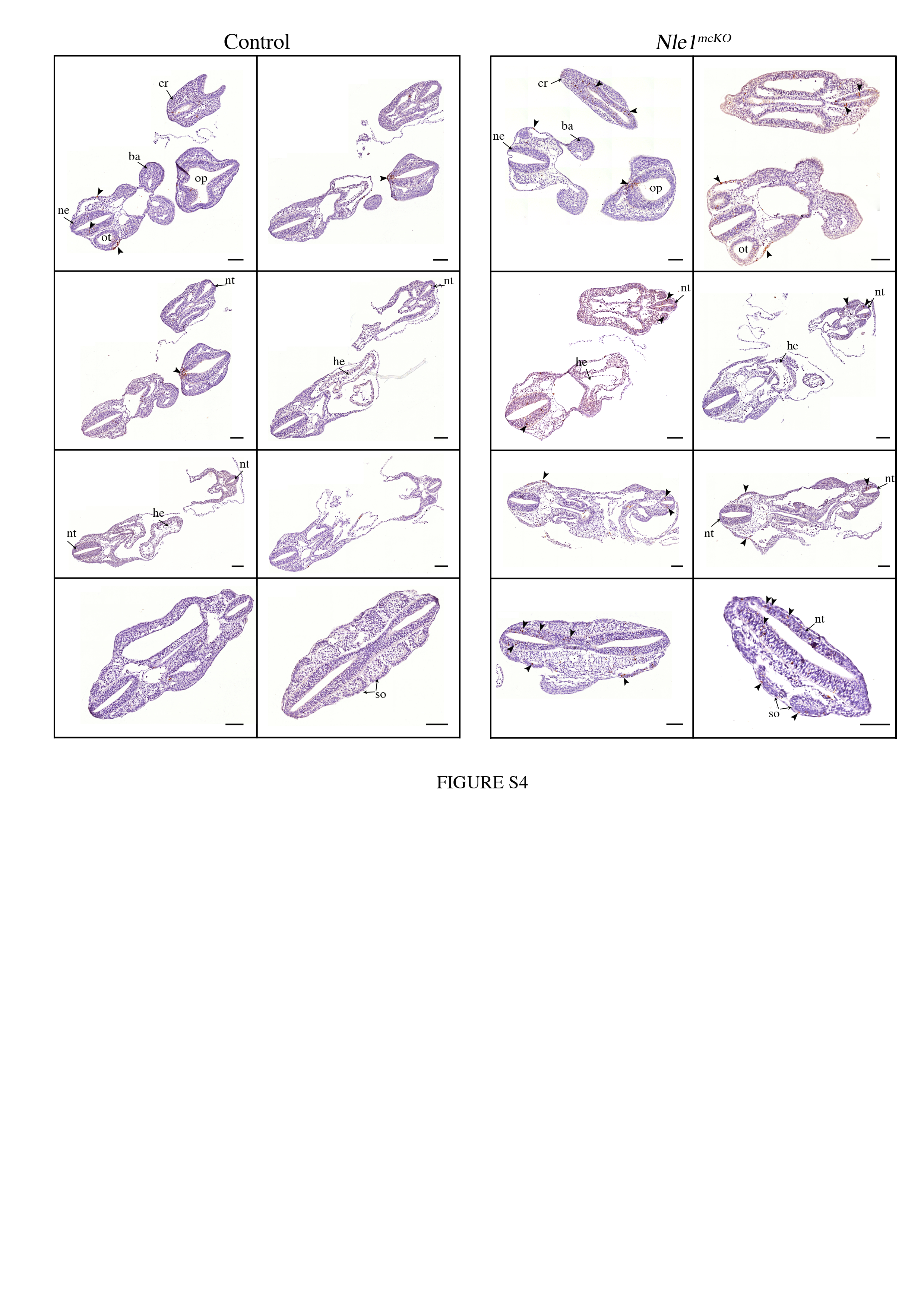

Supplement: Figure S4 — Analysis of apoptosis in E9.5 control and Nle1mcKO embryos. Immunostaining for the active form of caspase3 protein at E9.5 in control (left) and Nle1mcKO (right) embryos are shown. Embryos were embedded in agarose before being embedded in paraffin. Serial transverse sections are shown. In control embryos, apoptotic cells (arrowhead) were observed in the epidermis, the neural tube and otic pit. In Nle1mcKO embryos, an abnormally high number of apoptotic cells was observed in the neural tube caudally to the forelimb and in the epithelial and mature somites. Black arrowheads indicate upregulated apoptosis and red arrowheads indicate normal developmental apoptosis (n = 3 mutants and n = 4 controls including a Meox2Cre embryo). ba: branchial arch, cr: caudal region of embryo, he: heart, ne: neuroepithelium, nt: neural tube, ot: otic vesicle, op: optic vesicle, so: somite. (TIF) [file pone.0098507.s004.tif]
