# Supplementary material for: Notchless Is Required for Axial Skeleton Formation in Mice
Source: PLoS One. 2014 May 29;9(5):e98507. doi: 10.1371/journal.pone.0098507 (PMC4038589; doi:10.1371/journal.pone.0098507)
Supplement: Table S1 — Number of mutant embryos obtained from crosses between Nle1flox/flox and Nle1 Δ/+; Sox2Cre/+ mice at various embryonic stages. (DOCX) [file pone.0098507.s005.docx]

**Table S1.** Number of mutant embryos obtained from crosses between *Nle1^flox/flox^* and *Nle1*^Δ/+^*; Sox2^Cre/+^* mice at various embryonic stages.

| Embryonic stages | Total embryos | Mutant embryos^a^ |
| --- | --- | --- |
| E6.5 | 38 | 10 (26%) |
| E7.5 | 18 | 2 (11%) |
| E8.5-E9.5 | 20 | 0 |

^a^Absolute number and frequency (%).
